# Specific fibre composition and metabolism of the *rectus abdominis *muscle of bovine Charolais cattle

**DOI:** 10.1186/1471-2091-11-12

**Published:** 2010-03-05

**Authors:** Marie-Pierre Oury, Rollande Dumont, Catherine Jurie, Jean-François Hocquette, Brigitte Picard

**Affiliations:** 1 Etablissement National d'Enseignement Supérieur Agronomique de Dijon (ENESAD), BP 87999, 21079 Dijon Cedex, France; 2 Institut National de la Recherche Agronomique (INRA), Unité de Recherche sur les Herbivores UR 1213, 63122 St-Genès-Champanelle, France

## Abstract

**Background:**

An important variability of contractile and metabolic properties between muscles has been highlighted. In the literature, the majority of studies on beef sensorial quality concerns M. *longissimus thoracis*. M. *rectus abdominis *(RA) is easy to sample without huge carcass depreciation and may appear as an alternative to M. *longissimus thoracis *for fast and routine physicochemical analysis. It was considered interesting to assess the muscle fibres of *M. rectus abdominis *in comparison with *M. longissimus thoracis *(LT) and *M. triceps brachii *(TB) on the basis of metabolic and contractile properties, area and myosin heavy chain isoforms (MyHC) proportions. Immuno-histochemical, histochemical, histological and enzymological techniques were used. This research concerned two populations of Charolais cattle: RA was compared to TB in a population of 19 steers while RA was compared to LT in a population of 153 heifers.

**Results:**

RA muscle had higher mean fibre areas (3350 μm^2 ^*vs *2142 to 2639 μm^2^) than the two other muscles. In RA muscle, the slow-oxidative fibres were the largest (3957 μm^2^) and the fast-glycolytic the smallest (2868 μm^2^). The reverse was observed in TB muscle (1725 and 2436 μm^2 ^respectively). In RA muscle, the distinction between fast-oxidative-glycolytic and fast-glycolytic fibres appeared difficult or impossible to establish, unlike in the other muscles. Consequently the classification based on ATPase and SDH activities seemed inappropriate, since the FOG fibres presented rather low SDH activity in this muscle in comparison to the other muscles of the carcass. RA muscle had a higher proportion of I fibres than TB and LT muscles, balanced by a lower proportion either of IIX fibres (in comparison to TB muscle) or of IIA fibres (in comparison to LT muscle). However, both oxidative and glycolytic enzyme activities were lower in RA than in TB muscle, although the LDH/ICDH ratio was higher in RA muscle (522 *vs *340). Oxidative enzyme activities were higher in RA than in LT muscle, whereas glycolytic enzyme activity was lower. In RA muscle, contractile and metabolic properties appeared to be less well-correlated than in the two other muscles.

**Conclusions:**

RA muscle has some particularities in comparison to the LT and TB muscles, especially concerning the unusual large cross-section surface of SO fibres and the very low oxidative activity of intermediate IIA fibres.

## Background

Post-mortem meat tenderisation processes involve enzymatic and physicochemical mechanisms which are governed in intensity and amplitude by the proportion of the different fibre types [1].

Until 2004, three fibre types were distinguished in adult cattle muscles. In 1960, the fibre classification was based on metabolism (oxidative or glycolytic) [2]. In 1972, the classification of PETER *et al. *[3] based both on their contractile (ATPase activity) and metabolic properties (Succinate Dehydrogenase activity) divided fibres into slow oxidative (SO), fast oxidative-glycolytic (FOG) and fast glycolytic (FG) types. The revelation of myosin ATPase activity [4], or the use of specific antibodies of different myosin heavy chains isoforms (MyHC) [5] revealing only the contractile properties, allowed I, IIA and IIX fibres to be distinguished. More recently IIB fibres were identified in cattle [6-8]. Indeed, until 2004, many arguments existed to support the existence of myosin heavy chain (MyHC) isoform IIb in cattle but this isoform could not be formally identified. One explanation may be the negative correlation that appears in mammals between the concentration of MyHC IIb isoform and the size of the animals [6]. In addition, the presence of a common antigenic determinant between MyHC IIb and MyHC IIx contributed to confuse these two isoforms [7]. So for a long time, IIX fibres were classified as IIB fibres, because the techniques used did not allow these two isoforms to be distinguished [8]. The gene coding for the IIb myosin heavy chain has also been revealed in skeletal muscle of cattle [9]. However the presence of the protein was observed only in *extraocular *and *rectractor bulbi *cattle muscles [10,11]. Recently the study by Picard and Cassar-Malek [12] revealed the presence of a fourth MyHC isoform in *semitendinosus *and *longissimus thoracis *muscles of eleven 15-month-old Blonde d'Aquitaine young bulls. Western-blot analyses confirmed the presence of a fast MyHC. Altogether, these results indicate that a MyHC IIb isoform is expressed in the *semitendinosus *muscle and, in smaller amounts, in the *longissimus thoracis *muscle of some cattle. However, its expression does not seem to be frequent.

In addition to these pure fibres, the use of antibodies by immuno-histochemistry allows hybrid fibres to be distinguished, that contain multiple MyHC isoforms. There are in particular IIC fibres containing both MyHC I and MyHC IIa isoforms, or IIAX fibres containing MyHC IIa and MyHC IIx isoforms. On excessively large samples for which the histological techniques cannot be applied, the contractile and metabolic properties of muscles might also be estimated by electrophoresis using a homogenate and/or by measurement of enzyme activities [for review, [13]]. The most commonly used enzymes are on the one hand lactate dehydrogenase and phosphofructokinase for the glycolytic metabolism and, on the other hand, isocitrate dehydrogenase, citrate synthase or cytochrome-c-oxidase for the oxidative metabolism [14]. The different enzyme activities allow the muscles to be classified according to their metabolic type.

Considerable variability of contractile and metabolic properties between muscles might be highlighted. Indeed, the continuously active muscles like M. *diaphragrama*, M. *masseter *or the heart have a more oxidative metabolism than the less active muscles, which tend rather to have a glycolytic metabolism [15].

M. *rectus abdominis *(flank steak) is a muscle easy to sample without huge carcass depreciation. So this muscle might appear as an alternative to M. *longissimus thoracis *for fast and routine physicochemical analysis for studies on beef quality.

As part of two protocols established in our laboratory, observations were made of the specificities of the RA muscle.

The objective of this paper, by compiling these two studies, is to characterize the muscle fibres of RA muscle in comparison to two other muscles of the carcass, chosen in order to represent different anatomical regions, divergent growth patterns and functionalities. First RA muscle was compared to M. *triceps brachii *(TB) that is a muscle involved in movement and with M. *longissimus thoracis *(LT), a dynamic muscle (very active in the spine extension) that is often considered as a reference for which many results are available [16,17].

JURIE *et al. *[18] observed that between 15 and 24 months, the proportion of the slow oxidative fibers SO increases by 11% whereas the proportion of the fast oxido-glycolytic fibers FOG decreases. These evolutions lead to a more oxidative metabolism with age in the muscles of young bulls. Nevertheless, neither the proportions of the various types of fibers, nor the enzymatic activities (LDH and ICDH) appeared significantly modified by the age between cull cows of 4 and 9 years [19]. Thus, we found interesting to evaluate the samples of muscles at the commonly used age depending on animal type: 26 months when considering steers and 33 months when considering heifers.

## Methods

### Animals

The first study concerned the RA and TB muscles of 19 Charolais steers, slaughtered at 26 months of age and 405 kg of carcass weight. The muscles were sampled one hour *post mortem *at the INRA experimental slaughterhouse (Theix, France) [20].

The second study concerned the RA and LT muscles of 153 Charolais heifers, slaughtered at 33 months of age and 381 kg of carcass weight. The muscles were sampled 24 hours *post mortem *in an industrial slaughterhouse [21]. Thus, no one of these studies required an ethical approval.

### Measurements and analysis

#### Muscle samples

**Figure 1:**
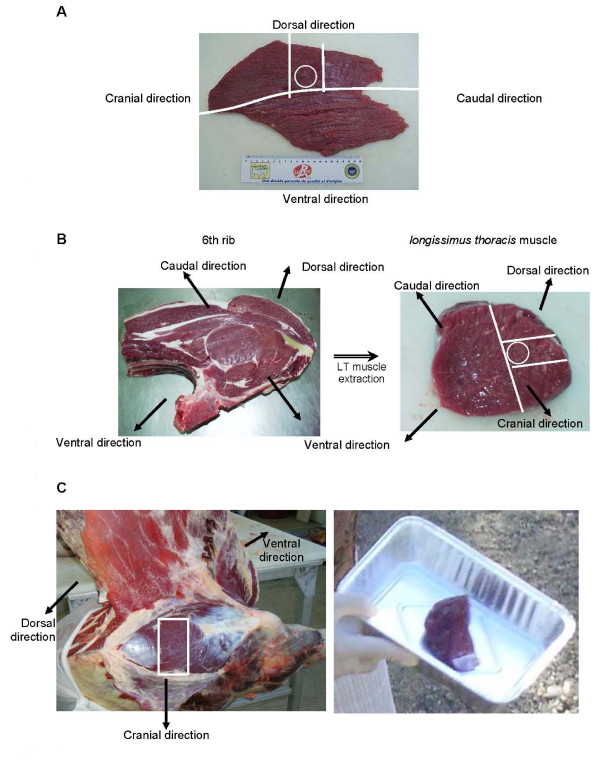
**Localisation of meat samples on each muscle**. A : RA muscle - *the sample was taken in the dorsal part of the muscle, in the middle, between cranial and caudal directions*. B : LT muscle - *the sample was taken in the 6^th^rib of the right half-carcass. The sample was taken in the middle of the muscle, between cranial and caudal directions*. C : TB muscle - *the sample was taken in the left half-carcass, in the middle of the muscle, directly under the skin.*

A standardization of the sampling procedures was adopted for each muscle with a clearly identified sample location (Figure 1). Samples intended for histochemical and immuno-histochemical analysis were cut into 5 mm side cubes and stacked on a cork in order to position the muscular fibres and to allow freezing. The freezing was made progressively into isopentane [22] then into liquid nitrogen and the samples were packaged at -80°C until analysis.

Samples intended for electrophoretic separations and metabolic properties of muscles were cut into small fragments, frozen in liquid nitrogen and packaged at -80°C before grinding.

#### Histochemical analysis: myofibrillar ATPase activity and metabolic properties

Delineation of the different types of fibres on serial cross-sections of muscles was based on the combination of the pH sensitivity of myofibrillar ATPase after acid pre-incubation at pH 4.2 [23] and succinate dehydrogenase (SDH) activity [24]. Myofibrillar ATPasic activity allows the distinction to be made between slow and fast fibres, because ATPase of slow fibres is stable in an acidic environment, and labile in a basic environment. The reverse situation is true for fast fibres. SDH activity allows the distinction to be made between oxidative and glycolytic fibres. This enzyme, characteristic of oxidative metabolism, is present in mitochondria. After revelation of SDH activity, oxidative fibres were coloured dark blue while glycolytic ones were coloured in pale blue. The combination of these two revelations enabled the different types of fibres to be identified as slow oxidative (SO), fast oxidative glycolytic (FOG) or fast glycolytic (FG) [3]. The percentage of each type of fibre was measured in two randomly selected areas on serial sections with an image analysis software program (Visilog). An average of 200 fibres were analysed in each serial section.

#### Fibre cross-sectional area

Fibre cross-sectional area was determined by computerized image-analysis on 10-μm thick sections cut with a cryotome MICROM HM 500 M at -25°C [5]. The sections were stained with azorubine to define the histological architecture of the muscle and to measure fibre proportion and diameter. For each muscle sample, we analyzed two different muscle sections located in the same sampling region. In each, there were in average 100 fibres, connective tissues and lipids. As fibers had not the same areas depending on animals and muscles, when considering the two different muscle sections, between 180 and 220 fibres at all were used to determine the fibre cross-sectional areas using computerized image-analysis. The surface area of each type of fibre and the mean fibre area were measured with the Visilog software program.

#### Immunohistochemical analysis of MyHC

Delineation of contractile types of fibres on serial cross-sections of muscle was based on the combination of three monoclonal antibodies specific to myosin heavy chain isoforms [5,25]: S5 15F4 specific for fast MyHC IIa and MyHC IIx isoforms, F 36 5B9 specific for slow MyHC I, and S5 8H2 specific for MyHC I and MyHC IIx (Table 1). Immuno-histochemistry allowed also identification of pure (I, IIA, IIX) and hybrid fibres (IIC with MyHC I and IIa, IIAX with MyHC IIa and IIx) [26].

**Table 1:** Fibres recognized by the different antibodies

ACm S5 15F4 IIA and IIX (fast fibres)	ACm F36 5B9 I (slow fibres)	ACm S5 8H2 I and IIX	Fibres
- - -	+++	+++	I

+++	- - -	+++	IIX

+++	- - -	- - -	IIA

+++	-	++	IIAX

++	++	+	IIC

+++ Fibers highly recognized by the antibody

++ Fibers well recognized by the antibody

+ Fibers slightly recognized by the antibody

- Fibers very slightly recognized by the antibody

- - - Fibers absolutely not recognized by the antibody

The specificity of the three anti MyHC antibodies used for this classification has been well described in bovine muscles by PICARD *et al. *[5]. The S5 8H2 antibody recognizes all fibres except IIA [5]. The comparisons of the three labels with each antibody on serial sections allow distinguishing: pure slow fibres labeled with F36 5B9 and S5 8H2 antibodies with high intensity; pure IIA fibres labeled only with S5 15F4 antibody and pure IIX fibres labeled with S5 15F4 and S5 8H2 but not with F36 5B9 (Table 1). We also observe fibres called hybrid fibres which contain different isoforms of MyHC. The IIC fibres are labeled with the three antibodies with low intensity, they contain I and IIa MyHC. The IIAX fibres are labeled with both S5 15F4 and S5 8H2 antibodies with low intensity. They contain IIa and IIx MyHC. This classification for bovine muscles with these antibodies has been published [5] and is used routinely in the lab. In adult animals as in this study the numbers of hybrid fibres is low, however in young animals or in foetuses, these three antibodies were used to distinguish these hybrid fibres and look at their development during foetal [27].

#### Electrophoresis of MyHC

The different types of myosin heavy chain isoforms were determined on the basis of previously determined migration pattern [28,29] using sodium Dodecyl Sulfate polyacrylamide gel electrophoresis (SDS-PAGE).

**Figure 2:**
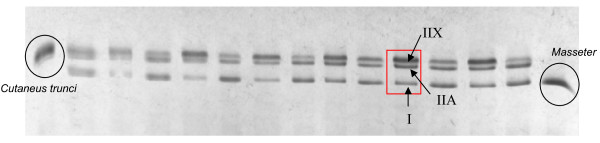
**Electrophoresis containing 13 samples of RA muscles and 2 standards**. The standards were the cutaneus trunci muscle, containing only fast fibres IIA and IIX and the masseter muscle containing only slow fibres I.

The bands were identified as MHC IIx, IIa, I on the basis of previously determined migration patterns [28,30]. Sodium Dodecyl Sulfate polyacrylamide gel electrophoresis (SDS-PAGE) was performed using the Laemmli method [31]. The stacking gel was 3.5% polyacrylamide with a concentration of 125 mM of Tris 1 M pH 6.8. The separating gel was a 5-8% polyacrylamide gradient with a crosslink of 1.96% and a concentration of 250 mM of Tris pH 8.8. The electrophoretic run was carried out at a constant voltage of 70 V for 2 h, followed by 130 V for 24 h at a temperature of 4°C. After migration, the gels were stained in a solution of R250 Coomassie Blue. The proteins were fixed in a solution made with ethanol (30%) and acetic acid (5%) for 20 min at room temperature. Then, gels were incubated in colored solution containing propanol 2 (25%), acetic acid (10%) and Coomassie Blue R250 (2 g/L) for 20 min and analysed using a ChemiMajeur densitometer (Figure 2).

#### Muscle metabolism

The metabolic type of muscle was determined by measuring enzyme activities. Anaerobic glycolytic metabolism was assessed by lactate dehydrogenase (LDH) activity [32]. Aerobic oxidative metabolism was assessed by isocitrate dehydrogenase (ICDH) and cytochrome-c oxidase (COX) activities according to the BRIAND *et al. *[33] and PIOT *et al. *[14] methods.

### Statistical analysis

Statistical analysis of each of the two studies was made separately, as the differences between the two experiments were too important (delay between slaughtering and muscle sampling, type of animal, number of animals).

So variance analysis and mean multiple comparisons with one factor were carried out using the GLM procedure (general linear model) from SAS 9.1 [34]. Multiple comparisons of the adjusted means (LSMEANS) were carried out using the PDIFF option of the GLM procedure.

Correlation coefficients were calculated using Pearson's model (SAS v 9.1).

Principal component analysis (PCA) for meat quality traits and physicochemical characteristics was made using WINSTAT software. This statistical method based on the study of the covariances and the correlations between variables allows the dimensions of the data set, originally described with a far larger number of variables. PCA allows the calculation of new variables, called principal components, which account for the variability in the data. The information can then be described with fewer variables than originally present since the principal components are linear combinations of the original variables. The first principal component is the combination of variables that explains the greatest amount of variability in the data. The second principal and subsequent components describe the maximum amount of remaining variability and must be independent of (orthogonal to) the first principal component. The main steps in PCA have been described for previous applications in cattle [35,36]. Results are presented in a 2D projection graph where variables near each other at the periphery of the circle are positively correlated, orthogonal variables are independent and variables separated by 180° are negatively correlated. The closer to the periphery of the circle, the higher the coefficient of correlation between variables.

## Results

### Proportions of SO, FOG and FG fibres in RA and TB muscles (histochemical analysis)

**Figure 3:**
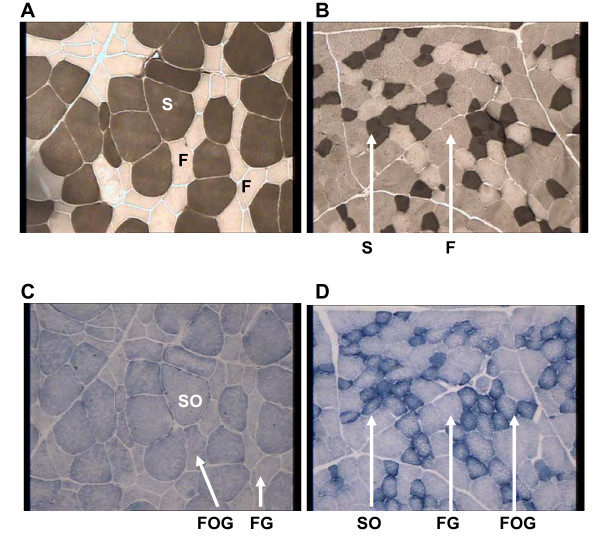
**Revelation of ATPase at pH 4.2 and succinate dehydrogenase (SDH) activities in RA and TB muscles of steers**. The width of each figure is 400 μm. A, C: RA muscle. B, D : TB muscle. A, B: revelation of ATPase activity at pH 4.2. The slow fibres (S) are coloured dark grey while fast ones (F) are coloured white or grey. C, D: revelation of Succinate DeHydrogenase activity. The oxidative fibres (SO) are coloured dark blue while glycolytic and oxidative-glycolytic ones (FG and FOG) are coloured pale blue. Scale of the cross-section : 600 μm (length)/400 μm (height).

In RA muscle, two different colours were observed when analysing ATPase activity revelation, black and white, which are characteristic of slow and fast fibres respectively. In TB muscle, the revelation allowed three different colours (black, grey and white) to be distinguished (Figure 3). In addition, the distinction between oxidative and glycolytic fibres in RA muscle was quite impossible, all fibres being pale blue. In TB muscle, revelation of the SDH activity easily allowed dark blue oxidative fibres and clear blue glycolytic ones to be distinguished (Figure 3).

The combination of ATPase and SDH classifications made it possible to distinguish SO, FOG and FG fibres. According to this method, RA muscle appeared to have significantly higher proportions of SO fibres (37.8 *vs *28.0%), offset by significantly lower proportions of FOG fibres (8.3 *vs *19.6%), as compared to TB muscle. The proportions of FG fibres were equivalent between the two muscles (53.9% in RA muscle and 52.4% in TB muscle; Table 2). Nevertheless, the contrasts between FG and FOG fibres were also less marked in RA muscle than in TB muscle.

**Table 2:** Muscular characteristics of TB and RA muscles from Charolais steers (first study)

	First study *(19 Charolais steers)*		
	**RA muscle**	**TB muscle**	**Significance:**
**Number of steers**	**19**	**19**	

**Different types of fibres according to histochemical analysis**			
SO fibre proportion (%)	37.8 (12)	28.0 (1.9)	**p < 0.001**
FOG fibre proportion (%)	8.3 (13)	19.6 (0.9)	**p < 0.001**
FG fibre proportion (%)	53.9 (13)	52.4 (1.9)	p > 0.10

**Fibre cross-sectional area according to histochemical analysis**			
SO	3957 (139)	1725 (359)	**p < 0.001**
FOG	3414 (105)	1961 (170)	**p = 0.003**
FG	2868 (103)	2436 (137)	**p = 0.041**
Mean	3324 (94)	2142 (168)	**p < 0.001**

**Different types of fibres according to immuno-histochemical analysis**			
Fibre I proportion (%)	40.4 (1.4)	27.6 (2.5)	**p < 0.001**
Fibre IIC proportion (%)	0.10 (0.05)	0.30 (0.20)	p > 0.10
Fibre IIA proportion (%)	30.7 (2.3)	23.9 (3.9)	**p = 0.021**
Fibre IIAX proportion (%)	5.4 (2.4)	5.8 (1.4)	p > 0.10
Fibre IIX proportion (%)	23.6 (1.9)	42.4 (4.7)	**p < 0.001**

**Myosin heavy chain (MyHC) isoforms *(SDS-PAGE electrophoresis)***			
MyHC I proportion (%)	38.5 (0.7)	20.7 (1.6)	**p = 0.003**
MyHC IIa proportion (%)	33.3 (0.7)	25.5 (2.4)	**p < 0.001**
MyHC IIx proportion (%)	28.2 (1.0)	53.8 (3.1)	**p < 0.001**

**Metabolic type of muscles *(enzyme activity determination)***			
ICDH activity (μmol.min.^-1^.g^-1^)	1.27 (0.05)	2.35 (0.06)	**p < 0.001**
LDH activity (μmol.min.^-1^.g^-1^)	617 (1.7)	753 (19)	**p < 0.001**
COX activity (μmol.min.^-1^.g^-1^)	7.6 (0.7)	11.0 (0.9)	**p = 0.003**
LDH/ICDH ratio	522 (15)	340 (28)	**p < 0.001**

means (standard error)

The classification of fibres in SO, FOG, FG on the basis of the revelation of ATPase and SDH activities on serial muscle sections is still used by different authors. The use of anti MyHC antibodies is better, it is why the two approaches were developed. However, the revelation of SDH activity in RA sections demonstrated clearly the particularity of this muscle with very low intensity comparatively to the other muscles

### Fibre cross-sectional area

**Figure 4:**
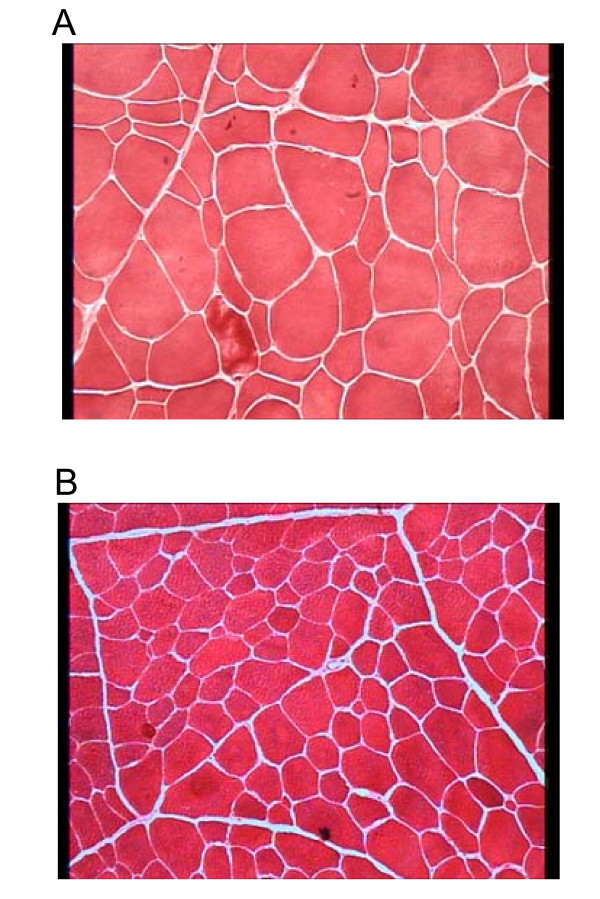
**Evaluation of mean fibre area by azorubine coloration in RA and TB muscles of steers**. The width of each figure is 400 μm. A,: RA muscle. B, : TB muscle. Muscular fibres are coloured red while connective tissue is coloured white. Scale of the cross-section : 600 μm (length)/400 μm (height).

RA muscle had muscular fibres with significantly higher mean surface area than those from TB (3324 *vs *2142 μm^2^; first study) and LT muscles (3379 *vs *2639 μm^2^; second study) (Tables 2 and 3; Figure 4).

**Table 3:** Muscular characteristics of LT and RA muscles from Charolais heifers (second study)

	Second study *(153 Charolais heifers)*		
	**RA muscle**	**LT muscle**	**Significance:**
**Number of heifers**	**153**	**153**	

**Fibre cross-sectional area *(histochemical analysis)***			
Mean	3379 (100)	2639 (64)	**p < 0.001**

**Myosin heavy chain (MyHC) isoforms *(SDS-PAGE electrophoresis)***			
MyHC I proportion (%)	31.6 (0.5)	22.3 (0.6)	**p < 0.001**
MyHC IIa proportion (%)	36.9 (0.7)	47.4 (1.2)	**p < 0.001**
MyHC IIx proportion (%)	31.1 (0.7)	29.7 (1.4)	p > 0.10

**Metabolic type of muscles *(enzyme activity determination)***			
ICDH activity (μmol.min.^-1^.g^-1^)	1.46 (0.04)	1.25 (0.04)	**p < 0.001**
LDH activity (μmol.min.^-1^.g^-1^)	555 (6)	835 (13)	**p < 0.001**
COX activity (μmol.min.^-1^.g^-1^)	16.6 (0.4)	10.4 (0.3)	**p < 0.001**
LDH/ICDH ratio	407 (57)	856 (108)	**p < 0.001**

means (standard error)

According to the first study, the higher mean fibre cross-sectional area in RA muscle was due to higher SO (3957 *vs *1725 μm^2^), FOG (3414 *vs *1961 μm^2^) and FG (2868 *vs *2436 μm^2^) fibre cross-sectional areas than in TB muscle (Table 2). In addition, SO fibres were the largest in RA muscle (3957 μm^2^) while they were the smallest in TB muscle (1725 μm^2^) (Figure 4). Conversely, in RA muscle, FG fibres were the smallest (2868 μm^2^) while they were the largest in TB muscle (2436 μm^2^). In both muscles, FOG fibres had an intermediate surface area.

### Proportions of I, IIA and IIX fibres (Immuno-histochemical analysis)

**Figure 5:**
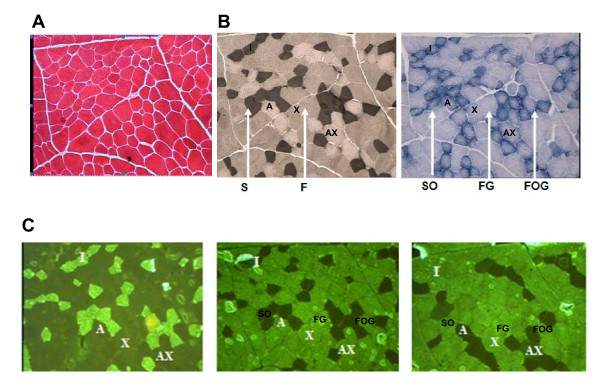
**Azorubine coloration, ATPase and SDH activities revelation and immunohistochemical analysis results on a identical serial cross-section of TB muscle**. A. Azorubine coloration. B. ATPase and SDH activities. C. Immunohistochemical results obtained with F365B9, S515F4 and S58H2 antibodies. Scale of the cross-section : 600 μm (length)/400 μm (height).

Immuno-histochemical analysis was made in parallel to histochemical analysis, only in the first study (Figure 5). RA muscle had significantly higher proportions of type I (40.4 *vs *27.6%) and IIA (30.7 *vs *23.9%) fibre than TB muscle, offset by significantly lower IIX fibre proportions (23.6 vs 42.4%). The hybrid IIC and IIAX fibre proportions were very limited (5.4 to 5.8 and 0.1 to 0.3% respectively) and equivalent between the two muscles (Table 2).

### Myosin heavy chain (MyHC) isoforms

According to the first study, RA muscle had significantly higher proportions of MyHC I (38.5 *vs *20.7%) and MyHC IIa (33.3 *vs *25.5%) isoforms than TB muscle, offset by significantly lower proportions of MyHC IIx isoform (28.2 *vs *53.8%; Table 2).

According to the second study, RA muscle had also significantly higher MyHC I isoform proportions than LT muscle (31.6 *vs *22.3%). This difference was offset by significantly lower proportions of MyHC IIa isoform (36.9 *vs *47.4%). The proportions of MyHC IIx isoform were equivalent between RA and LT muscles (31.1 and 29.7% respectively; Table 3). Indeed, 13.7% of LT muscle samples had no MyHC IIx isoform (n = 21/153), whereas only 1.3% of RA muscle samples did not contain this isoform (n = 2/153).

### Metabolic type of muscles

Metabolic activities were not compared between the two studies due to different sampling procedures but the overall differences between muscle types were similar.

Enzyme activities for ICDH (1.27 *vs *2.35 μmol.min.^-1^.g^-1^), COX (7.6 *vs *11.0 μmol.min.^-1^.g^-1^) and LDH (617 *vs *753 μmol.min.^-1^.g^-1^) were significantly lower in RA muscle than in TB muscle, reflecting a lower metabolic activity in RA muscle (first study; Table 2). The ratio between LDH and ICDH activities was significantly different between the two muscles, being higher for RA muscle (522 *vs *340; p < 0.001) than TB muscle.

The ICDH (1.46 *vs *1.25 μmol.min.^-1^.g^-1^) and the COX (16.6 *vs *10.4 μmol.min.^-1^.g^-1^) oxidative enzyme activities were significantly higher in RA muscle than in LT muscle (second study). In contrast, the activity of LDH glycolytic enzyme was significantly lower in RA muscle (555 *vs *835 μmol.min.^-1^.g^-1^; Table 3). The ratio between LDH and ICDH activities was significantly lower for RA muscle (407 *vs *856; p < 0.001) than for LT muscle.

### Correlation coefficients between certain muscle properties/multivariate analysis

**Figure 6:**
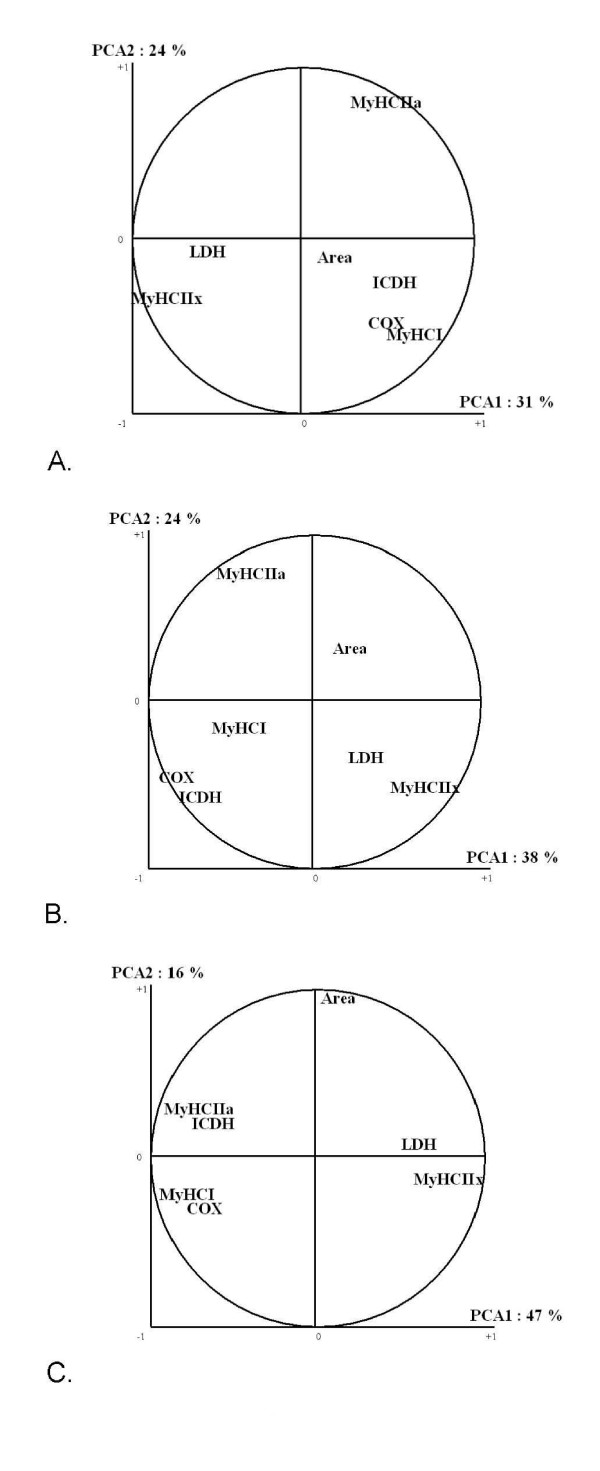
**Principal Component Analysis on muscular properties in RA, TB and LT muscles**. A: RA muscles of 153 Charolais heifers. B : TB muscles of 19 Charolais steers. C: LT muscles of 153 Charolais heifers. PCA 1, PCA 2 : proportion of variance explained by axes 1 and 2. The PCA was established only on the muscular properties studied in the three muscles; MyHC I, IIa, IIx : proportions of myosin heavy chain isoforms I, IIa and IIx; LDH: Lactate Dehydrogenase activity; ICDH: Isocitrate Dehydrogenase activity; COX: Cytochrome-c-Oxidase activity; Area : mean fibre cross-sectional area.

The relationships between muscle properties were studied by principal component analysis (PCA) for each of the three muscles separately. The dataset obtained for each muscle was subjected to multivariate analyses to summarize each muscle according to its contractile and metabolic properties (Figure 6). The ability of these features to discriminate contractile and metabolic properties was examined by canonical discriminant analyses. Almost 55% of the data variance was explained by the first two factors. The contractile and metabolic properties were slightly more closely related in LT and TB muscles than in RA since the two major principal component axes explained respectively 62, 63 and 55% of the total variability. The first principal component axis was predominantly explained by the variability in muscle metabolism. It opposed the oxidative characteristics (MyHC I, ICDH, COX) to glycolytic ones (MyHC IIx and LDH). In RA and TB muscles, the second principal component axis was explained by the myosin isoform of oxidative-glycolytic fibres (MyHC IIa) whereas in LT muscle, it was explained by mean fibre cross-sectional area. As previously indicated by MORENO *et al. *[37], the three major fibre type populations (I, IIA and IIX) were clearly discriminated.

MyHC I proportions were positively correlated with ICDH and COX activities, but the correlations were significantly higher in LT (r = +0.43 and +0.54) and TB muscles (r = +0.62 and +0.50) than in RA muscle (r = +0.31 and +0.37). Moreover, the negative correlation between MyHC I proportions and LDH activity was less marked in RA muscle (r = -0.22; p = 0.04) than in TB and LT muscle (r = -0.39 and -0.45; p < 0.001 respectively). In RA muscle, MyHC IIx proportions were not significantly correlated with LDH and COX activity, but were negatively correlated with ICDH activity (r = -0.23; p = 0.04). In TB and LT muscles, MyHC IIx proportions were significantly correlated with the three metabolic enzyme activities. On the one hand MyHC IIx was found to be positively correlated with LDH activity, while on the other hand MyHC IIx was found to be negatively correlated with ICDH and COX activities. COX and ICDH activities were positively correlated to each other (r between +0.27 and +0.59; p < 0.01). No significant correlation was found between MyHC IIa proportions and oxidative enzyme activities in RA muscle, whereas they were significant in TB and LT muscles.

## Discussion

### Muscle fibre cross-sectional areas

RA muscle mean fibre cross-sectional area was significantly much higher than those from LT and TB muscles, so is clearly a specificity of this muscle. To our knowledge, no previous research has established the mean fibre cross-sectional area of RA muscle. However, LT muscle seems to be characterized by small fibre cross-sectional area in comparison to other muscles such as M. *semitendinosus *(ST) [38].

In RA muscle, the FG fibres were the smallest whereas the SO fibres were the biggest. These observations contrast with those found for muscles usually studied, such as LT or ST, where SO or I fibres present the smallest surface area and FG or IIX fibres the biggest [39,40]. When studying the thigh cross-section of 34 of the largest muscles of bovine, the proportion of type I fibres appeared highest in the anterior and medial parts, while the IIX fibres tended to be concentrated in the superficial and posterior parts [39]. Nevertheless, mean fibre cross-sectional area does not change with fibre location.

#### Muscle fibre type

According to histochemical analysis, RA muscle had a higher proportion of oxidative SO fibres than TB muscle. The proportions of oxidative-glycolytic FOG fibres appeared to be lower in RA muscleand those of FG fibres were found to be equivalent in the two muscles. According to the literature, significant differences are detected among fibre types for SDH histochemical activities. Some authors indicated that SDH mean activities tended to decrease significantly from SO to FG fibres [37] whereas other [41] revealed significant changes in fibre size within a single muscle. Nevertheless, in RA muscle, the revelation of SDH activity on serial sections appeared to be low and the oxidative fibres were also hardly separated from glycolytic ones. Moreover, ATPase activity in RA muscle allowed slow (S) and fast (FOG and FG together) fibres to be separated, but not FOG and FG fibres. Consequently in this muscle the proportion of FOG fibres is underestimated. In TB muscle, the SO, FOG and FG fibres were easily made out. Because of the low revelations afforded by ATPase and SDH activities, some FOG fibres may have been classified as FG in RA muscle. The proportion of FOG and FG fibres could also have been under/over-estimated. Thus one original result of this study is the demonstration that in RA muscle, a classification based on ATPase and SDH activities is not appropriate, since FOG fibres have no SDH activity and are probably confused with FG fibres.

The hypothesis of under-estimation of FOG proportions and over-estimation of those of FG in RA muscle is corroborated by the evaluation of contractile type of fibre and myosin heavy chain isoforms proportions. With immuno-histochemical analysis, RA muscle appeared to have higher proportions of I and IIA fibres, but lower proportions of IIX fibres than TB muscle. An equivalent conclusion might be obtained with the evaluation of myosin heavy chain isoforms proportions, where the proportions of MyHC IIx isoforms are significantly lower in RA muscle than in LT muscle.

The two classifications are not exactly the same [5,42], but the I, IIA and IIX fibres may respectively be compared to SO, FOG and FG fibres [3]. In TB muscle, the proportions of FOG fibres, IIA fibres and MyHC IIa isoforms were quite similar (19.6, 23.9 and 25.5% respectively). The proportions of FG fibres, IIX fibres and MyHC IIx isoforms were also quite similar in this same muscle (52.4, 42.4 and 53.8% respectively). However in RA muscle, immuno-histochemistry and myosin isoform proportions gave the same results but histochemical analysis indicated lower oxidative-glycolytic (FOG) and higher glycolytic (FG) fibre proportions than expected. So in this muscle, the proportion of IIA fibres is not correlated with that of FOG fibres. The proportions of fibre types and the significant differences between the methods led to the conclusion that histochemistry is not appropriate to quantify FOG and FG fast fibres in this muscle. This indicates that in RA muscle fibres expressing MyHC IIa have a very low oxidative activity which is not observable with the revelation of SDH activity on muscle sections. It demonstrates clearly that the classification of PETER *et al. *[3] based on the revelation on serial sections of ATPase and SDH activities cannot be used in RA muscle. For slow fibre type the different methods give the same proportions of in RA muscle different methods give the same proportions in RA muscle (from 37.8 to 40.4%), as already seen by PICARD *et al. *[5]. Indeed, the proportion of slow fibres (I, SO and MyHC I) of RA muscle was not significantly modified whatever the method used and the three methods may also be suitable. The close results obtained when studying contractile and metabolic type of fibres in RA, LT and TB muscles seems logical. Indeed, these fibres represent a homogeneous population, being slow in a oxidative metabolism, as already indicated by PICARD *et al. *[5].

As seen before, the histochemical results have to be considered with caution, because of the low SDH and ATPase activities that do not allow the distinction to be made between these two types of fast fibres. Three reasons may explain the previous observation. First the FOG fibres may be less oxidative in RA muscle than in other muscles, secondly RA muscle may have a smaller mitochondria content, and thirdly the enzymatic activity may be lower in RA muscle.

#### Muscle metabolic activity

Lower metabolic activities have been verified as RA muscle had lower enzymatic activities than TB muscle, both concerning the oxidative (ICDH and COX) and the glycolytic (LDH) metabolisms, as already seen by TALMANT *et al. *[43]. RA muscle metabolism appeared less active than that of TB muscle, maybe because of the location of these two muscles in the carcass. Thus, some specificity either in the number or in the activity of mitochondria in RA muscle needs to be verified. As the ratio between the glycolytic and the oxidative metabolisms is significantly higher in RA muscle, this indicates that this muscle is less oxidative than TB muscle. This is in agreement with the previous conclusions of CASSAR-MALEK *et al. *[44], who indicated that RA and TB muscles were oxidative-glycolytic and oxidative respectively. Based on analysis of a set of five biochemical variables generally measured for the assessment of muscle type, OUALI *et al. *[45] indicated that RA and TB muscles can be included neither in slow oxidative nor in fast-glycolytic muscles and have also to be classified as intermediate.

When comparing RA and LT muscles, ICDH and COX activities were found to be higher and LDH activity was found to be lower for RA muscle. These results may be linked with the higher proportion of oxidative MyHC I and the lower proportion of MyHC IIa fibres in RA muscle. These properties confirmed the less glycolytic and more oxidative metabolism in RA than in LT muscle. OUALI *et al. *[45] suggested that LT muscle might be classified as fast glycolytic, using the same set of five biochemical variables as previously indicated.

No significant correlation was found between MyHC IIa proportions and oxidative enzyme activities in RA muscle, whereas they were significant in TB and LT muscles. According to PICARD *et al. *[5], IIA fibres, that contain MyHC IIa isoforms, might be separated in LT muscle into oxidative and non-oxidative IIA fibres depending on their metabolic properties. ARIANO *et al. *[46] also described two subclasses of FOG fibres. However, in RA muscle, the distinction between these two types of IIA fibres, but also between fibres FOG and FG, is disturbed by a very low fast fibre SDH activities [8], leading to less oxidative MyHC IIa isoforms in RA than in the LT muscle. This conclusion corroborated the hypothesis that FOG fibres may be less oxidative in RA muscle than in other muscles. It could be supposed that some MyHC IIa isoforms don't present an oxidative but a glycolytic metabolism. Indeed, this is the case for some muscles and in particular for LT muscle. The PCA carried out on the contractile and metabolic properties of this muscle confirmed this hypothesis: in LT muscle, there is a very strong correlation between ICDH and COX activities, MyHC IIa and MyHC I isoform proportions. This correlation, less marked in TB muscle, may explain the difference between IIA and FOG fibre proportions in this muscle (23.9 *vs *19.6%).

## Conclusions

RA muscle appeared to have some particularities in comparison to two muscles commonly used to characterize meat, such as LT and TB muscles. These particularities concern especially mean fibre cross sectional area, and particularly type I fibres which have the higher areas, whereas the IIX fibres have the lower ones. This hierarchical organization is generally opposite in the other muscles of the carcass. Moreover in RA, IIA fibres have a low oxidative activity, consequently they can not be classified by histochemistry with the revelation of SDH activity. Further researches are in progress to explain these specificities of this muscle, especially with respect to the number and the metabolism of mitochondria in FOG fibres.

The implications of RA specificities on meat quality traits appeared contradictory. On the one hand, the higher mean fibre areas could be unfavourable to meat quality traits. Indeed, previous results indicated a negative correlation between tenderness and mean fiber area in M. *longissimus thoracis *(r ranging from -0.33 to -0.39) [47,48]. On the second hand, oxidative enzyme activities were higher in RA than in LT muscle, whereas glycolytic enzyme activity was lower and RA had a higher proportion of I fibres than TB and LT muscles. These specificities could be in favour of meat tenderness. Indeed, in M. *longissimus thoracis*, M. *semitendinosus *and M. *triceps brachii*, tenderness scores increased and shear force on broiled meat decreased with muscle oxidative metabolism (MyHC I proportion and COX activity). The more oxidative muscles were of higher quality, particularly in terms of tenderness [48]. Moreover, some previous publications indicated that muscles with a high proportion of slow oxidative fibers or a low proportion of fast glycolytic fibers were more tender [49-51]. Studies in process are about to establish the impact of *rectus abdominis *specificities on meat quality traits, in comparison to other muscles.

To finish with, it may be interesting to include this type of muscle when characterizing muscles metabolism in a carcass, as its properties are quite different from those of the other muscles.

## Authors' contributions

MPO: muscle sample collection, data collection, statistical analysis, manuscript preparation; RD: conception, manuscript preparation, critical contribution to the final manuscript; CJ: statistical analysis, critical contribution to the final manuscript; JFH: critical revision of the manuscript, substantial contribution to the final manuscript; BP: design, muscle sample collection, substantial contribution to the final manuscript. All authors read and approved the final manuscript

## References

[B1] Ouali A Meat tenderization: possible causes and mechanisms Journal of Muscle Foods 1990 1 129 165 10.1111/j.1745-4573.1990.tb00360.x

[B2] Dubowitz V A comparative histochemical study of oxidative enzyme and phosphorylase activity in skeletal muscle Histochemie 1960 2 105 117 10.1007/BF00744575 13724667

[B3] Peter JB Barnard RJ Edgerton VR Gillespie CA Stemple KE Metabolic profiles of three fibre types of skeletal muscle in guinea pigs and rabbits Biochemistry 1972 11 2627 2633 10.1021/bi00764a013 4261555

[B4] Brooke MH Kaiser KK Muscle fibre types : how many and what kind? Arch Neurol 1970 23 369 379 4248905 10.1001/archneur.1970.00480280083010

[B5] Picard B Duris MP Jurie C Classification of bovine muscle fibres by different histochemical techniques Histochemical Journal 1998 30 473 479 10.1023/A:1003207922944 10192530

[B6] Hamalaïnen N Pette D Patterns of myosin isoforms in mammalian skeletal muscle fibres Crosc Res Technol 1995 30 381 389 10.1002/jemt.1070300505 7787237

[B7] Schiaffino S Gorza L Satore S Saggin L Ausoni S Vianello M Gundersen K Lomo T Three myosin heavy chain isoforms in type 2 skeletal muscle fibres Journal of Muscle Research and Cellular Motility 1989 10 195 205 10.1007/BF01739810 2547831

[B8] Picard B Jurie C Cassar-Malek I Hocquette JF Typologie et ontogenèse des fibres musculaires chez le bovin INRA Productions Animales 2003 16 125 131

[B9] Chikuni K Muroya S Nakajima I Myosin heavy chain isoforms expressed in bovine skeletal muscles Meat Science 2004 67 87 94 10.1016/j.meatsci.2003.09.011 22061120

[B10] Maccatrozzo L Patruno M Toniolo L Reggiani C Mascarello F Myosin heavy chain 2B isoform is expressed in specialized eye muscles but not in trunk and limb muscles of cattle Eur J Histochem 2004 49 357 366 15718201

[B11] Mascarello J Stechinni ML Rowlerson A Ballochi E Tertiary myotubes in postnatal growing pig muscle detected by their myosin isoform composition Anim Sci 1992 70 1806 1813 10.2527/1992.7061806x 1386067

[B12] Picard B Jurie C Cassar-Malek I Is the myosin heavy chain IIb isoform expressed in bovine muscles? Arch Tierz 2006 49 Special 91

[B13] Hocquette JF Ortigues-Marty I Damon M Herpin P Geay Y Métabolisme énergétique des muscles squelettiques chez les animaux producteurs de viande INRA Productions Animales 2000 13 185 200

[B14] Piot C Veerkamp JH Bauchart D Hocquette JF Contribution of mitochrondria and peroxisomes to palmitate oxidation in rat and bovine tissues Comparative Biochemistry and Physiology 1998 121 69 78 10.1016/s0305-0491(98)10087-1 9972294

[B15] Hocquette JF Graulet B Olivecrona T Lipoprotein lipase activity and mRNA levels in bovine tissues Comparative Biochemistry and Physiology Brevue 1998 121 85 96 10.1016/s0305-0491(98)10090-1 9972295

[B16] Shackelford SD Koohmaraie M Wheeler TL Effects of slaughter age on meat tenderness and USDA carcass maturity scores of beef females Journal of Animal Science 1995 73 3304 3309 8586588 10.2527/1995.73113304x

[B17] Johnston DJ Reverter A Robinson DL Ferguson DM Sources of variation in mechanical shear force measures of tenderness in beef from tropically adapted genotypes, effects of data editing and their implications for genetic parameter estimation Austr J Exp Agric 2001 41 991 996 10.1071/EA00018

[B18] Jurie C Bauchart D Culioli J Dransfield E Jailler R Lepetit J Listrat A Martin JF Ouali A Geay Y Picard B Modifications des caractéristiques du muscle Longissimus Thoracis chez les taurillons entre 15 et 24 mois Rencontres, Recherche, Ruminants 2002 9 265

[B19] Jurie C Bauchart D Culioli J Dransfield E Jailler R Lepetit J Listrat A Martin JF Ouali A Geay Y Picard B Les caractéristiques du muscle Longissimus Thoracis ne sont pas modifiées chez les vaches de réforme entre 4et 9 ans d'âge Rencontres, Recherche, Ruminants 2002 9 266

[B20] Micol D Oury MP Picard B Hocquette JF Briand M Dumont R Egal D Jailler R Dubroeucq H Agabriel J Effect of age at castration on animal performances, muscle characteristics and meat quality traits in 26-month-old Charolais steers Livestock Science 2008 120 116 126 10.1016/j.livsci.2008.05.002

[B21] Oury MP Agabriel J Agabriel C Micol D Picard B Blanquet JP Roux M Dumont R Relationship between rearing practices and eating quality traits of the muscle *rectus abdominis *of Charolais heifers Livestock Science 2007 111 242 254 10.1016/j.livsci.2007.01.154

[B22] Bevilacqua A Zaritzky NE Calvelo A Histological measurements of ice in frozen beef Journal of Food Technology 1979 14 237 251

[B23] Guth L Samaha FJ Qualitative differences between actomyosin ATPase of slow and fast mammalian muscle Exp Neurol 1969 25 138 152 10.1016/0014-4886(69)90077-6 4241609

[B24] Pearse AGE Chruchill JA Histochemistry theorical and applied 1968 London: England 948 950

[B25] Jurie C Nedelec J Picard B Production des anticorps monoclonaux spécifiques des chaînes lourdes de myosine INRA Productions Animales 1998 11 146 149

[B26] Pons F Leger J Chevallay M Tome F Fardeau M Leger J Immunocytochemical analysis of myosin heavy chains in human fetal skeletal muscles J Neurol Sci 1986 76 151 163 10.1016/0022-510X(86)90165-6 3540217

[B27] Picard B Jurie C Duris MP Renand G Consequences of selection for higher growth rate on muscle fibre properties in cattle Livestock Production Sciences 2005 102 107 120 10.1016/j.livsci.2005.12.001

[B28] Young OA Davey L Electrophoretic analysis of proteins from single bovine muscle fibres Biochemical Journal 1981 195 317 327 6458285 10.1042/bj1950317 PMC1162888

[B29] Picard B Robelin J Geay Y Influence of castration and postnatal energy restriction on the contractile and metabolic characteristics of bovine muscle Ann Zootech 1995 44 347 357 10.1051/animres:19950402

[B30] Picard B Barboiron C Duris MP Gagniere H Jurie C Geay Y Electrophoretic separation of bovine muscle myosin heavy chain isoforms Meat Science 1999 553 1 7 10.1016/S0309-1740(99)00021-2 22062926

[B31] Laemmli UK Cleavage of structural proteins during the assembly of the head of the bacteriophage TA Nature 1970 227 680 685 10.1038/227680a0 5432063

[B32] Ansay M Individualité musculaire chez le bovin : étude de l'équipement enzymatique de quelques muscles Ann Biol Anim Biochim Biophys 1974 14 471 486 10.1051/rnd:19740308

[B33] Briand M Tamant A Briand Y Monin G Durand B Metabolic types of muscle in the sheep: I myosin ATPase glycolytic and mitochondrial enzyme activities Eur J Appl Physiol 1981 46 347 358 10.1007/BF00422122 6455290

[B34] SAS Institute Guide for Personal Computers Version 91 Cary 2002 NC : SAS Institute

[B35] Hocquette JF Bas P Bauchart D Vermorel M Geay Y Fat partitioning and biochemical characteristics of fatty tissues in relation to plasma metabolites and hormones in normal and double-muscled young growing bulls Comparative Biochemistry and Physiology A 1999 122 127 138 Erratum,. Comparative Biochemistry and Physiology A 1999, **123 **: 311-312. 10.1016/s1095-6433(98)10172-1 10216937

[B36] Cassar-Malek I Picard B Kahl S Hocquette JF Relationships between thyroid status, tissue oxidative metabolism, and muscle differentiation in bovine fetuses Domestic Animal Endocrinology 2007 33 1 91 106 10.1016/j.domaniend.2006.04.011 16797912

[B37] Moreno-Sanchez N Diaz C Carabano MJ Rueda J Rivero JLL A comprehensive characterisation of the fibre composition and properties of a limb (Flexor digitorum superficialis, membri thoraci) and a trunk (Psoas major) muscle in cattle BMC Cell Biology 2009 9 67 19077313 10.1186/1471-2121-9-67 PMC2630315

[B38] Jurie C Martin JF Listrat A Jailler R Culioli J Picard B Effects of age and breed of beef bulls on growth parameters, carcass and muscle characteristics Animal Science 2005 80 257 263 10.1079/ASC40710257

[B39] Totland GK Kryvi H Distribution patterns of muscle fibre types in major muscles of the bull (*Bos taurus*) Anatomy and Embryology 1991 184 441 450 10.1007/BF01236050 1835822

[B40] Picard B Lefaucheur L Berri C Duclos MJ Muscle fibre ontogenesis in farm animal species Reproduction Nutrition Development 2002 42 415 431 10.1051/rnd:2002035 12537254

[B41] Rivero JL Galisteo AM Aguera Skeletal muscle histochemistry in male and female Am J Vet Res 1993 1993 54 675 680 10.1016/0034-5288(93)90051-g 8460254

[B42] Staron RS Pette D Correlation betweeen myofibrillar ATPase activity and myosin heavy chain composition in rabbit muscle fibres Histochemestry 1986 86 19 23 10.1007/BF00492341 2432036

[B43] Talmant A Monin G Briand M Dadet M Briand Y Activities of metabolic and contractile enzymes in 18 bovine muscles Meat Science 1986 18 23 40 10.1016/0309-1740(86)90064-1 22055463

[B44] Cassar-Malek I Hocquette JF Jurie C Listrat A Jailler R Bauchart D Briand Y Picard B Muscle-specific metabolic, histochemical and biochemical responses to a nutritionally induced discontinuous growth path Animal Science 2004 204 79 59

[B45] Ouali A Sentandreu MA Aubry L Boudjellal A Tassy C Geesink GH Farias-Maffet G Hocquette JF, Gigli S Meat toughness as affected by muscle type Indicators of milk and beef quality 2005 Wageningen: The Netherlands 447 451

[B46] Ariano MA Amstrong RB Edgerton VR Hindlimb muscle fibre populations of five mammals Journal of Histochemical Cytochemistry 1973 21 51 55 10.1177/21.1.51 4348494

[B47] Seideman SC Methods of expressing collagen characteristics and their relationship to meat tenderness and muscle fiber types Journal of Food Science 1986 51 273 276 10.1111/j.1365-2621.1986.tb11107.x

[B48] Renand G Picard B Touraille C Berge P Lepetit J Relationships between muscle characteristics and meat quality traits of young Charolais bulls Meat Science 2001 59 49 60 10.1016/S0309-1740(01)00051-1 22062505

[B49] Dransfield E Martin JF Bauchart D Abouelkaram S Lepetit J Culioli J Jurie C Picard B Meat quality and composition of three muscles from French cull cows and young bulls Animal Science 2003 76 387 399

[B50] Ockerman HW Jaworek D Van Stavern B Parrett N Pierson CJ Castration and sire effects on carcass traits, meat palatability and muscle fiber characteristics in Angus cattle Journal of Animal Science 1984 59 981 990

[B51] Strydom PE Naude RT Smith MF Scholtz MM Van Wyk JB Characterisation of indigenous African cattle breeds in relation to meat quality traits Meat Science 2000 55 79 88 10.1016/S0309-1740(99)00128-X 22060907

